# The Endocrine Disruption of Prenatal Phthalate Exposure in Mother and Offspring

**DOI:** 10.3389/fpubh.2020.00366

**Published:** 2020-08-28

**Authors:** Yiyu Qian, Hailing Shao, Xinxin Ying, Wenle Huang, Ying Hua

**Affiliations:** Department of Obstetrics and Gynecology, The Second Affiliated Hospital of Wenzhou Medical University, Wenzhou, China

**Keywords:** phthalate, endocrine disruptors, prenatal exposure, birth outcomes, pregnancy outcomes

## Abstract

Phthalates are a group of ubiquitous synthetic endocrine-disrupting chemicals. Fetal and neonatal periods are particularly susceptible to endocrine disorders, which prenatal exposure to phthalates causes. There is increasing evidence concerning the potential endocrine disrupting for phthalate exposure during pregnancy. This article aims to review the endocrine impairment and potential outcomes of prenatal phthalate exposure. Prenatal exposure phthalates would disrupt the levels of thyroid, sex hormone, and 25-hydroxyvitamin D in pregnant women or offspring, which results in preterm birth, preeclampsia, maternal glucose disorders, infant cryptorchidism, infant hypospadias, and shorter anogenital distance in newborns, as well as growth restriction not only in infants but also in early adolescence and childhood. The relationship of prenatal phthalate exposure with maternal and neonatal outcomes in human beings was often sex-specific associations. Because of the potentially harmful influence of prenatal phthalate exposure, steps should be taken to prevent or reduce phthalate exposure during pregnancy.

## Introduction

Phthalates are a family of endocrine-disrupting chemicals broadly used as plasticizers in various industrial commodities (1). Phthalates would be gradually released from their substrate materials and be accumulated at a measurable concentration in different environments such as atmosphere, fresh water, sediments, soils, and landfills because of their refractory characteristics. Σdi-2(ethylhexyl)-phthalate (DEHP) and Σdibutyl phthalate (DBP) are the primary phthalate ester pollutants in the environment (2, 3). For example, DEHP has been detected in atmospheric particulate matter, fresh water and sediments, soil, and landfills with 0.54–689 ng/m^3^, not detected (N.D.) to 197 μg/L, N.D. to 34,800 μg/kg dry weight, and N.D. to 63,000 μg/kg dry weight, and N.D. to 232.50 μg/L, respectively (2). Then, they would be delivered to the human body and accumulated through diet, inhalation, and skin contact and rapidly transforming into even more toxic metabolites (2–4). According to the molecular weight, the metabolites of phthalate are divided into two groups: low-molecular-weight phthalates (LMWPs) and higher-molecular-weight phthalates (HMWP). Both groups of phthalates and their major metabolites are shown in Table 1. The metabolites of phthalate can cross the placenta and be detected in placental tissue, amniotic fluid, cord blood, and neonatal meconium (5) and participate in embryo development and growth.

**Table 1:** Phthalates and their major metabolites.

**Phthalate**	**Primary metabolite**	**Secondary metabolite**
**Low-molecular-weight (LMW) phthalates**		
DMP: dimethyl-phthalate	MMP: monomethyl-phthalate	
DEP: diethyl-phthalate	MEP: monoethyl-phthalate	
DiBP: di-iso-butyl-phthalate	MiBP: mono-iso-butyl-phthalate	MHiBP or 2OH-MiBP: mono 2-hydroxy-isobutyl phthalate
DnBP: di-n-butyl-phthalate	MnBP: mono-n-butyl-phthalate	MHBP or 3OH-MnBP: mono 3-hydroxybutyl phthalate
DBP: dibutyl phthalate	MBP: mono-butyl phthalate	
**High-molecular-weight (HMW) phthalates**		
BBzP: butyl benzyl phthalate	MBzP: monobenzyl-phthalate	MCPP: mono-3-carboxypropyl-phthalate
DiNP: diisononyl-phthalate	MiNP: monoisononyl-phthalate	MHiNP or OH-MiNP: mono(hydroxyisononyl)-phthalate
		MOiNP or oxo-MiNP: mono(oxoisononyl)-phthalate
		MCiOP, MCOP, or cx-MiNP: mono(carboxyisooctyl)- phthalate
DiDP: diisodecyl-phthalate	MiDP: monoisodecyl-phthalate	MOiDP or oxo-MiDP: mono(oxoisodecyl)-phthalate
		MHiDP or OH-MiDP: mono(hydroxyisodecyl)-phthalate
		MCiNP, MCNP or cx-MiDP: mono(carboxyisononyl)- phthalate
**di-2(ethylhexyl)-phthalate (DEHP)**		
DEHP: di-2(ethylhexyl)-phthalate	MEHP: mono(2-ethylhexyl)-phthalate	MEHHP or 5OH-MEHP: mono(2-ethyl-5-hydroxyhexyl)- phthalate
		MEOHP or 5oxo-MEHP: mono(2-ethyl-5-oxohexyl)- phthalate
		MECPP or 5cx-MEPP: mono(2-ethyl-5-carboxypentyl)- phthalate
		MCMHP: mono(2-carboxyhexyl) phthalate

Pregnancy is a critical period for fetal development when exposed to phthalates, which can lead to serious and even permanent adverse effects of infants and children (6). Although underlying molecular mechanisms of health effects related to phthalate exposure during gestation are poorly understood, animal studies indicated that phthalate exposure can impair the function of Leydig cells, which disrupt testicular steroidogenesis, sperm quality, and fertility (7). It had been reported that phthalate metabolites could activate the estrogen receptor (ER) α and ERβ, androgen receptor (AR), peroxisome proliferator-activated receptor α (PPARα), and proliferator-activated receptor γ (PPARγ) *in vitro* (8, 9), which play crucial roles in inflammation, metabolism, and other disease processes (10, 11). Besides, di(isononyl)cyclohexane-1,2-dicarboxylate (DINCH), a non-phthalate plasticizer, has taken the place of phthalate due to the lower migration rate and toxicity (12). Although deemed safe by classical regulatory toxicological standards (13), DINCH and its metabolite cyclohexane-1,2-dicarboxylic acid monoisononylester would activate human ERα, ERβ, AR, PPARα, and PPARγ (14) and then affect disease processes (10, 11). Because of the limited quantity of research on the effect of DINCH and its metabolite on pregnant women, this review did not cover the literature about DINCH and its metabolite.

**Figure 1:**
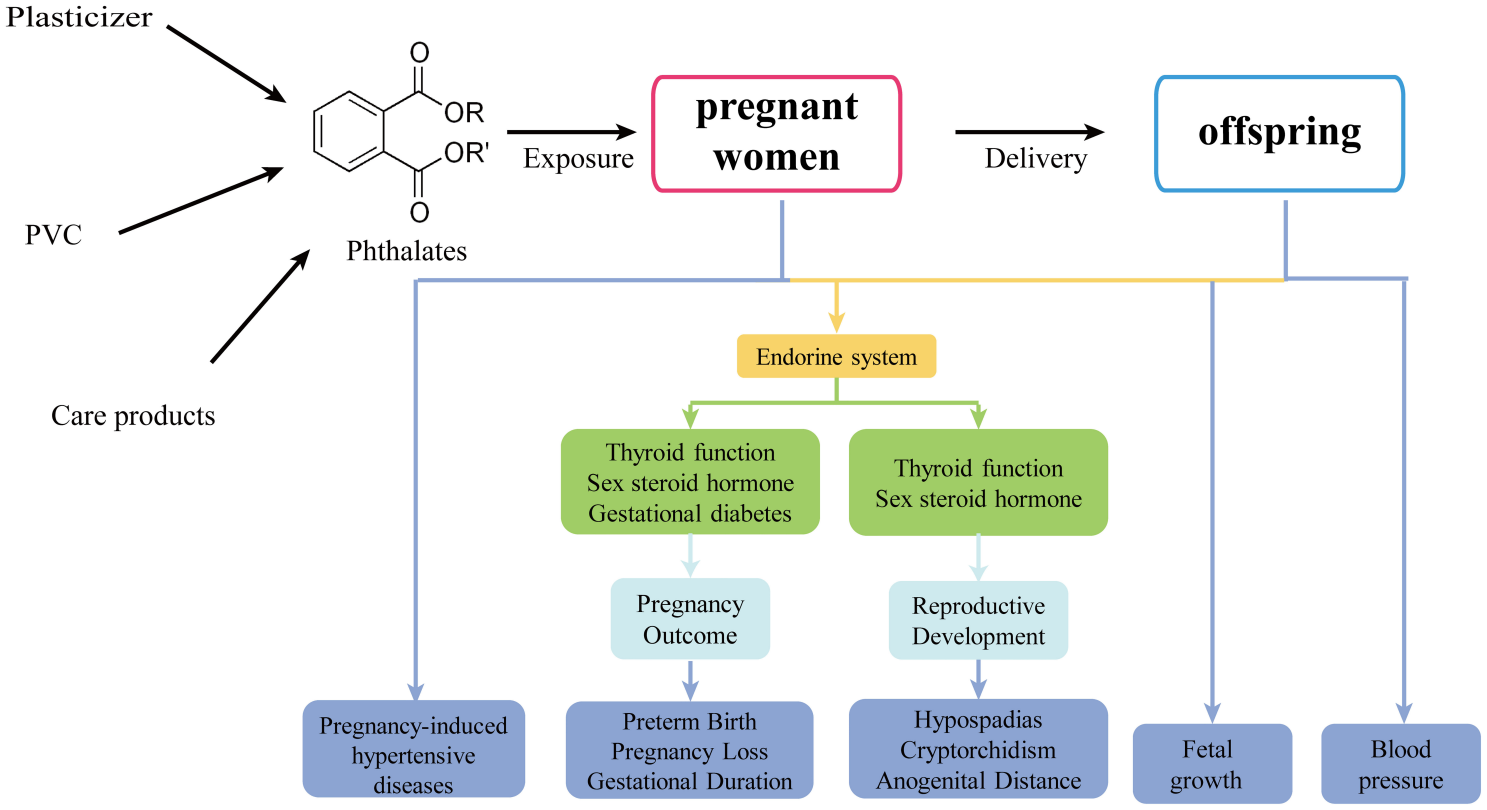
The effects of prenatal phthalate exposure on pregnant women and offspring.

As phthalates ubiquitous presence in the environment, increasing evidence focused on the potential negative health effects of exposure to phthalate during pregnancy. This article provides an overview of the maternal exposure to phthalates and its endocrine impairments on women and offspring, including the alterations of thyroid and sex hormone levels, genital abnormalities, gestational diabetes mellitus (GDM), hypertensive diseases, vitamin D homeostasis, fetal and child's growth restriction, abortion, and preterm labor (Figure 1).

## Prenatal Phthalate Exposure and Hormone Homeostasis

Homeostasis hormones are essential for pregnancy maintenance and fetal development. Recently, a growing body of researches provided evidence that maternal phthalate exposure was toxic to thyroid and reproductive system (15–37), which may be a biological process between phthalates and impairments. Besides, the effects on the fetus, neonate, or child are varied by infant's sex (Table 2).

**Table 2:** The effect of prenatal phthalate exposure on hormone homeostasis.

**Negative association**	**Positive association**	**References**
**Maternal serum thyroid hormones**		
MBP with T4 and FT4		(15)
MnBP with T4	MiBP with T4	(16)
MBzP, MEHP, and MEHHP with T4 and FT4	MBzP, MEHP, and MEHHP with TSH	(17)
MCPP and MCOP with FT3 ΣDEHP with FT4	MiBP with FT4	(18)
DEHPm with TSH	DEHPm with T4, FT4, and T3	(19)
MBP and MEHP with TT4		(20)
MnBP with T4 DEHPm, MEOHP, and MECCP with TSH MEHHP, MEOHP, MECCP, and DEHPm with T3	MCMHP with T4 MMP MiBP, MnBP, and ΣDBPm with T3 MEP and MiBP with FT4	(21)
	MBP, MCOP, MCPP, and MHBP with FT4 MBP, MCOP, and MCPP with T4 MBP, MBzP, MCOP, MCPP, MECPP, MEHHP, MEOHP, MiBP, MHBP, MHiBP, and MONP with T3	(22)
MEP with TT4		(23)
**Cord serum thyroid hormones**		
–	–	(17)
Generally no (except MBzP with TSH)	–	(24)
Generally no (except MBzP with TSH)	–	(23)
	ΣDBPm with T3 and FT4	(21)
**Child serum thyroid hormones**		
	MEHP with FT4	(25)
**Maternal sex hormones**		
–	–	(15)
DEHPm and MCNP with FT	MiBP, MBzP, and MEOHP with estrone and E2	(26)
DEHPm, MBP, and MBzP with FT and TT in women with females	MBP, MBzP, and MEP with TT and FT in women with males	(27)
MEP with P4 and SHBG		(18)
MEHP with SHBG DiNPm with DHEA-S and androstenedione	MECPP and MCiOP with SHBG	(28)
MCNP, MCPP, MECPP, MEHHP, and MEOHP with CRH MEP with testosterone MCOP with SHBG MCOP with P4	MHBP with testosterone	(22)
**Fetal and neonatal sex hormones**		
MEP, MEHP, 5oxo-MEHP, and 5OH-MEHP with FT and FT/E2 in females		(29)
MEHP with T/E2, P4, and inhibin B in males		(30)
Σphthalates and MiBP with DHEA and androstenedione MnBP with testosterone	MnBp with E2 MnBP with Σphthalates and E3	(21)
	MEHP with DHEA, ratios of DHEA/androstenedione	(32)
	5cx-MEPP with androstenedione and T	(33)
**Child's sex hormones**		
	MiBP and MEP with T DEHPm (MEHP, MECPP, MEHHP, and MEOHP), MBP, MiBP, and MCPP with DHEA-S	(6)
No with serum estradiol and SHBG	MBzP, MEP nd T DEHPm (MECPP, MEHHP, and MEOHP), MEHP with DHEA-S MEHP and MECPP with inhibin B	(34)
Phthalate metabolites with DHEA-S	MEHP and DEHPm with SHBG MBzP and MEP with T	(35)
Phthalate metabolites with DHEAS and inhibin B	MEOHP, MBzP, MBP, MCPP with SHBG	(36)
MEP with estradiol MBzP with DHEA-S	ΣDEHP with estradiol MBzP, MCPP and ΣDBP with SHBG	
MEHP with LH/T	MiNP with FSH the sum of MCMHP and MECPP with LH	(37)
**Maternal Vitamin D homeostasis**		
DEHP metabolites with MCPP with total 25(OH)D	DEHP metabolites with vitamin D deficiency	(38)

### Prenatal Phthalate Exposure and Maternal Thyroid Hormones

Maternal thyroid dysfunction was associated with abnormal fetal development such as growth retardation, inadequate central nervous system development, preterm birth, and so on (39).

Huang et al. (15) first provided evidence that maternal thyroid activity would be impaired due to the exposure of phthalate during pregnancy. Maternal serum thyroxine (T4) and free thyroxine (FT4) concentrations were negatively associated with concentrations of urinary mono-butyl phthalate (MBP) during the second trimester (27.9 + 2.3 weeks) among 76 Taiwanese pregnant women (15). Moreover, Huang et al. (16) further found that serum T4 was negatively associated with urinary mono-n-butyl-phthalate (MnBP) during early pregnancy, but FT4 was positively associated with mono-iso-butyl-phthalate (MiBP). Yao et al. (17) detected that the urinary monobenzyl-phthalate (MBzP), mono(2-ethylhexyl)-phthalate (MEHP), and mono(2-ethyl-5-hydroxyhexyl)-phthalate (MEHHP) concentration were associated with low concentrations of maternal T4 and FT4 and were positively related to maternal TSH during ~10 gestational weeks. It is well-known that the fetal thyroid function is immaturity before 12 weeks of gestation and relies solely on the mother. These studies (15–17) suggested that prenatal phthalate exposure decreasing maternal T4 levels during pregnancy might be a potential risk to pregnancy maintenance.

Johns et al. (18) collected urine samples and serum samples at two study visits (18 ± 2 and 26 ± 2 gestational weeks) and found generally inverse relationships between phthalate exposure and free triiodothyronine (FT3) and FT4, except a positive association between MiBP and FT4 (18). And these inverse associations were stronger in about 26 weeks of gestation, especially for urinary mono-3-carboxypropyl-phthalate (MCPP) and mono(carboxyisooctyl)-phthalate (MCiOP) with serum FT3, and Σdi-2(ethylhexyl)-phthalate (DEHP) [the sum of MEHP, MEHHP, mono(2-ethyl-5-oxohexyl)-phthalate (MEOHP), and mono(2-ethyl-5-carboxypentyl)-phthalate (MECPP)] with FT4 (18). To investigate the magnitude of the potential thyroidal-disrupting effect of phthalate exposure in different periods of gestation, they performed up to four visits across pregnancy (10, 18, 26, and 35 weeks of gestation). Urinary DEHP metabolite concentrations were generally negatively related to TSH, and it has statistical significance at visits 1 and 2 (19). Besides, DEHP metabolites were positively associated with T4 and FT4 at visits 1 and 4 and with T3 at visit 1 (19). In a China cohort study, stratification analyses by gestational weeks (5–8, 9–12, and 13–20 gestational weeks) showed that the negative effects of MBP or MEHP on maternal total T4 (TT4) began as early as the embryonic stage of 5–8 gestational weeks (20). In another study, after stratification by study visit (median:18, 26, and 39 gestational weeks), maternal serum T4 levels were negatively associated with urinary MnBP levels but positively with urinary mono(2-carboxyhexyl) phthalate (MCMHP) levels, and FT4 were positively associated with monoethyl-phthalate (MEP) and MiBP at visit 1 (21). Several phthalate metabolites, such as MEOHP, MECPP, and DEHP metabolites, were inversely correlated with TSH at visit 2 and T3 at visit 3. Urinary levels of monomethyl-phthalate (MMP) at visit 1 and MiBP, MnBP, and DBP metabolites at visit 2 were positively associated with T3 levels (21). Contrary to previous studies, a recently ongoing cohort study reported the generally positive relationship between prenatal phthalate exposure with maternal thyroid hormones (THs), such as maternal urinary MBP, MCiOP, MCPP, and mono 3-hydroxybutyl phthalate (MHBP) concentrations with serum FT4, MBP, MCiOP, and MCPP with T4, as well as MBP, MBzP, MCiOP, MCPP, MECPP, MEHHP, MEOHP, MiBP, MHBP, monohydroxyisobutyl phthalate (MHiBP), and mono(oxoisononyl)-phthalate (MOiNP) and T3 (22).

However, in a recent prospective cohort, Romano et al. (23) reported that individual phthalate metabolites in maternal urine were generally not associated with changes in maternal serum TH, except increasing MEP was associated with decreased TT4. To date, it is still unclear whether exposure to phthalates and their metabolite during pregnancy would activate or inhibit thyroid functions. Moreover, it is difficult to determine the magnitude and/or direction of the relationships between certain phthalate exposure and maternal TH at different periods of pregnancy.

### Prenatal Phthalate Exposure and Fetal or Child's Thyroid Hormones

As phthalates could cross the placenta, prenatal phthalate exposure could impair fetal thyroid functions; moreover, the effect may persist until childhood. However, several studies suggest that individual maternal urinary phthalate metabolite concentrations have no bearing on cord serum THs (17, 23, 24), except MBzP was negatively associated with TSH in cord serum (23, 24). Conversely, the study done by Huang et al. (21) reported that maternal ΣDBPm levels at the second trimester (median = 26 weeks' gestation) were positively associated with T3 and FT4 levels in cord serum.

Moreover, the effect of prenatal phthalate exposure on thyroid function might last until the period of childhood. A study from Columbia Center showed that maternal urinary MEHP levels were positively associated with FT4 in children at age 3 years (25). The absolute change in the THs of fetal and child caused by prenatal phthalate exposure was small, but even small changes may impair fetal growth and development. So, it is urgent to elucidate the effects of prenatal exposure to phthalates on THs in offspring.

### Prenatal Phthalate Exposure and Maternal Sex Hormones

Despite one single study stating that phthalate metabolites in maternal urine were not correlated with maternal serum concentrations of progesterone (P4), estradiol (E2), and follicle-stimulating hormone (FSH) at the second trimester (15), most evidence suggests that prenatal phthalate exposure could alter reproductive hormone levels in the mother (18, 26–28).

In a study investigating the relationship between phthalate exposure with maternal sex hormones during early pregnancy, urinary MiBP, MBzP, and MEOHP concentrations were positively associated with estrone and E2 concentrations, whereas DEHPm and mono(carboxyisononyl)-phthalate (MCiNP) were inversely related to free testosterone (FT) concentrations (26). The results were less consistent in a recent study, in which maternal serum testosterone was positively associated with urinary MHBP concentrations, but negatively with MEP, and MCiOP was negative with P4 (22). Moreover, Sathyanarayana et al. (27) found that the relationships varied by fetal sex. The inverse associations that maternal urinary concentrations of MBP, MBzP, and sum of DEHP metabolites with log-FT and -TT concentration were statistically significant for women with female fetuses and the positive relationships between MEP, MBP, and MBzP with TT and FT were observed in women carrying male fetuses only (27).

A prospective study reported that maternal urinary MEP was strongly associated with low serum P4 across gestation and marginally significantly associated with lower serum sex hormone–binding globulin (SHBG) at about 18 weeks of gestation (18). Not only MEP, but also maternal MEHP concentrations were inversely associated with SHBG concentrations in an Australian cohort, and the sum of diisononyl-phthalate (DiNP) metabolites was negatively correlated with dehydroepiandrosterone sulfate (DHEA-S) and androstenedione at 18 weeks of gestation, although the phthalates were measured in maternal serum samples (28). However, the SHBG concentrations were positively correlated with MECPP and MCiOP concentrations at 36 weeks of gestation (28), as well as MCiOP across 18–26 weeks of gestation (22). In addition, they suggested that CRH, a critical role in the timing of delivery and preterm birth, was negatively associated with MCiNP, MCPP, MECPP, MEHHP, and MEOHP (22).

Taken together, those studies supported the hypothesis that phthalate exposure during pregnancy would alter maternal levels of sex hormone, and the timing of exposure during gestation plays a crucial role in the magnitude of the potential endocrine-disrupting effect of phthalates.

### Prenatal Phthalate Exposure and Fetal and Neonatal Sex Hormones

Reproductive system is the main targets of phthalates, especially in males. Multiple research studies have reported the associations between intrauterine phthalate exposure and reproductive hormones in cord blood (29–32) and amniotic fluid (33), which provided evidence that maternal phthalate exposure could affect fetal sex steroid hormones status. The explanation for this may be that phthalates disrupt the function of Leydig and Sertoli cells in testis (4).

A study including 155 mother-infant pairs from Taiwan suggests that the maternal urinary concentrations of phthalate metabolites at the third trimester, such as MEP, MEHP, and its metabolites (MEHHP and MEOHP), were inversely correlated with FT and FT/E2 of cord blood in females, whereas it was not significant in male newborns (29). On the contrary, a Japanese prospective cohort study reported that inverse associations between maternal serum MEHP levels during 23–35 weeks and concentrations of T/E2, P4, and inhibin B in cord blood were significant only for males (30).

The effects of maternal phthalate exposure on fetal reproductive hormones are conflicting. Maternal plasma phthalate levels during pregnancy in the third trimester were negatively correlated with cord blood DHEA, androstenedione, and testosterone, while positively associated with estradiol and estriol (31). However, associations between maternal serum MEHP levels and the high levels of DHEA and high ratios of DHEA/androstenedione in cord serum were reported later (32).

One Danish study assessed the effects of phthalates on steroid hormone levels in amniotic fluid. MECPP concentrations in amniotic fluid were related to higher levels of androstenedione and T in amniotic fluid (33). The conclusion was limited because the amniotic fluid biobank consisted of cryptorchidism and hypospadias and control boys. Taken together, these findings (30–33) confirmed that prenatal phthalate exposure would affect the levels of fetal sex hormone, while the sex-specific effect was difficult to evaluate.

### Prenatal Phthalate Exposure and Child's Sex Hormones

Recently, the long-term impairment of prenatal phthalate exposure on the reproductive endocrine of female (6, 34) and male (35–37) newborns has been of great concern. Several studies had reported the effect of prenatal phthalate exposures on the reproductive hormone in children at 8–14 years old or puberty.

A team from Mexico provided evidence that prenatal phthalate exposure could affect the sex hormone of peripubertal female children. The data showed that higher serum T concentrations during peripubescence of children were associated with concentrations of MBzP, MEP, and MiBP in maternal urine (6, 34). *In utero* MBP, MiBP, and MCPP, as well as DEHPm (MEHP, MECPP, MEHHP, and MEOHP), exposures were correlated with higher peripubertal DHEA-S concentrations, and MEHP and MECPP were both positively associated with inhibin B (6, 34).

For male children, another Mexican team reported that phthalate exposure during the third trimester of pregnancy was associated with reproductive hormones change in male children of 8–14 years old (35). For instance, maternal urinary phthalate metabolites were generally related to increased SHBG levels, especially MBzP, MBP, MEOHP, and MCPP, and were marginally inversely associated with DHEAS and inhibin B levels. Watkins et al. (36) further reported that prenatal phthalate exposure, specifically at the third trimester, was associated with an increase in serum SHBG concentrations and decreased odds of adrenarche rather than in early pregnancy. In addition, there were negative associations of MEP with estradiol and of MBzP with DHEA-S at mean 25.1 gestational weeks (36). These findings suggest that phthalate exposures during pregnancy may impact circulating levels of sex hormones in offspring during peripuberty and at the timing of sexual maturation, and the third trimester of *in utero* development may be the potential windows of phthalate exposure susceptibility (6, 34–36).

Hart et al. (37) assessed the reproductive function at 20 years of age with antenatal phthalate exposure in males. Positive associations were reported between antenatal serum monoisononyl-phthalate (MiNP) concentration in mother and adult serum FSH concentration after adjustment for body mass index (BMI). Besides, the maternal sum of DEHP metabolites (MCMHP and MECPP) was positively associated with serum LH in offspring, whereas MEHP, one of the DEHP metabolites, was negatively correlated with serum ratio of LH to testosterone (37).

In summary, prenatal phthalate exposure may impair reproductive development in early adolescence and have potential and long-term effects on child development. Because the case definitions, the analytical matrices, and the timing of exposure assessment were various, which may be the reason why the influence of phthalate exposure on reproductive hormone and sexual maturation during the pubertal transition was different.

## Prenatal Phthalate Exposure and Vitamin D Homeostasis

Vitamin D homeostasis not only plays a well-established role in skeletal health, but also is necessary for normal fetal growth and pregnancy outcomes during pregnancy. It was widely considered that phthalates are endocrine disruptors and could disrupt the balance of 25-hydroxyvitamin D (25(OH)D) in pregnant women.

Certain urinary phthalate metabolites, especially for DEHP metabolites and MCPP, were correlated to low circulating total 25(OH)D levels at median 10 and 26 gestational weeks, and the associations in white women were weaker than those in women of black or other race/ethnicity (38).

There were significant associations between DEHP metabolites and ~20% increase in the odds of vitamin D deficiency at median 10 weeks (38). Therefore, prenatal exposure to phthalates may reduce levels of circulating total 25(OH)D, which could result in adverse maternal and neonatal outcomes such as fetal growth restriction, preeclampsia, and spontaneous preterm labor (40, 41). Future research should verify these findings and illuminate the public health impact of phthalate exposure on circulating 25(OH)D of pregnant women.

## Prenatal Phthalate Exposure and Complication of Pregnancy

Recently, several groups assessed the effect of phthalate exposure on glycemia, hypertensive, and vitamin D homeostasis during pregnancy and hypothesized that the effect may associate with pregnancy complications, including GDM and pregnancy-induced hypertensive diseases (42–54). So it is important to characterize the potential risk of phthalate exposure during pregnancy.

### Prenatal Phthalate Exposure and Gestational Diabetes Mellitus

It was reported that phthalate exposure led to a loss and an abnormal ultrastructural pattern of β cells in the pancreas, thereby reducing the insulin content and altering blood glucose levels (55). To date, a lot of epidemiological research had evaluated the associations between maternal phthalate exposure and gestational glycemia levels (42–46), but there was not enough evidence to draw a definitive conclusion.

In a cohort study (*n* = 72), maternal urinary concentrations of MiBP and MBzP during the first trimester (median = 12.8 gestational weeks) were correlated to lower blood glucose levels at the time of GDM screening after adjustment for urinary creatinine, race/ethnicity, and gestational age (42). Using a prospective cohort design, Fisher et al. (43) observed that a positive association between MEHP, MCiOP, and MEHP/MECPP and 120-min glucose levels measured at 28 gestational weeks and apparent non-linear correlations between MiBP exposure and higher odds of GDM. And maternal MEP exposure was positively associated with odds of GDM, which was reported by a recent larger-population study (44). However, a larger prospective cohort study from Canada (*n* = 1,274) failed to observe the association between exposure to phthalates in first-trimester and impaired glucose tolerance (IGT) or GDM (45).

Additionally, James-Todd et al. (46) assessed the influence of phthalate exposure on GDM risk factors such as the BMI in the first trimester, gestational weight gain (GWG), and blood glucose levels in the second trimester. They found that MEP levels in maternal urine were positively associated with the odds of IGT and excess GWG, whereas higher concentrations of MnBP, MCPP, and ∑DEHP were associated with less excessive GWG, lower continuous blood glucose, and fewer IGT, respectively (46).

Taken together, these findings provide important information about the effect of phthalates on blood glucose levels during pregnancy. Further studies need to be performed with a variety of specimen types (including urine) and assessment of exposure at multiple time points.

### Prenatal Phthalate Exposure and Hypertensive Diseases

Hypertensive disorders complicating pregnancy, including gestational hypertension, preeclampsia, eclampsia, and HELLP (hemolysis, elevated liver enzyme levels, and low platelet levels) syndrome, is one of the leading causes of mortality and morbidity, which mechanism was multisystemic and heterogeneous, broadly classified on placental and maternal (47). Phthalates have been shown to restrict the placental labyrinth layer in rodents, which have a similar function of human syncytiotrophoblasts. And the reduction of syncytiotrophoblasts is associated with preeclampsia (56, 57). Another possible etiology for hypertensive diseases in humans is that phthalates may cause placenta proinflammatory and extensive oxidative stress via peroxisome PPARγ activation (58).

A recent study reported that MiBP concentrations in maternal urine during the first trimester were correlated to the increased diastolic blood pressure (BP) in the second trimester among the women with male fetuses (48). Moreover, evidence from an American study pointed to the positive effect of prenatal phthalate exposure on the onset of preeclampsia, they measured a maternal urinary phthalate at four visits (9.7, 17.9, 26.0, and 35.1 weeks' gestation) and defined preeclampsia as systolic BP ≥140 mmHg or diastolic BP ≥90 mmHg after 20 weeks' gestation along with positive urinary protein testing (49). They observed that the DEHP metabolite concentrations across pregnancy and MEP at about 9.7 weeks' gestation were positively associated with the odds of preeclampsia, especially for the mother with female fetuses (49). Besides, Werner et al. (50) observed that maternal urinary MBzP concentrations at 16 weeks of gestational age, not at 26 gestational weeks, were positively associated with maternal diastolic BP and risk of hypertensive disorders complicating pregnancy. One study, on the contrary, reported that no association between any phthalate metabolite exposure during pregnancy and gestational hypertensive disorders, except di n-octyl phthalate (DnOP), was associated with lower diastolic BP (51). Besides, in a multicenter clinical trial, MiBP concentrations in maternal urine samples were associated with a decrease both in systolic and diastolic BP, and MBP was associated with a decrease in systolic BP (52).

Two studies further assessed the long-term effect of prenatal phthalate exposure on children's BP. Valvi et al. (53) reported that the sum of HMWP metabolites and the sum of LMWP metabolites were associated with lower systolic BP *Z* scores at 4–7 years of age in girls but not in boys, while no significant association was shown with diastolic BP *Z* scores. However, a recent study reported that early-life phthalate exposure did not affect children's BP at ages 6–11 years (54).

To conclude, the evidence that phthalate exposure during pregnancy may influence maternal or child's BP was limited. More thorough studies are eager to evaluate how phthalate exposure impairs the BP of pregnant women and their child.

## Prenatal Phthalate Exposure and Pregnancy Loss

Pregnancy loss means the death of the embryo or fetus, including the loss of pregnancy of human chorionic gonadotropin elevation or the loss of a clinical pregnancy (59). Phthalate exposure may affect pregnancy maintenance and cause pregnancy loss because of the endocrine disruption. Most epidemiologic evidence showed phthalate exposure was related to higher odds of pregnancy loss not only in couples conceiving naturally (59–62) but also among women undergoing medically assisted reproduction (63) (Table 3).

**Table 3:** The effect of prenatal phthalate exposure on pregnancy loss and gestational age.

**Negative association**	**Positive association**	**Reference**
**Pregnancy loss**		
MEHP with clinical pregnancy loss	MEHP with subclinical embryonic loss	(59)
	MEP, MEOHP, MEHHP, and ΣHMWP with embryonic lossMEHHP with fetal loss	(60)
	MEHP, MEHHP, MEOHP, and the sum of DEHP metabolites with biochemical pregnancy loss or total pregnancy loss (<20 gestational weeks)	(63)
	MEHP and MMP with missed miscarriage (<20 gestational weeks)	(61)
	MEP, MiBP, and MEHP with clinical pregnancy loss	(62)
	the sum of MiBP and MnBP with recurrent pregnancy loss	(64)
	MiBP and MEHP with unexplained recurrent spontaneous abortion	(65)
	DEHP with missed abortion	(66)
MEOHP with early pregnancy loss (<6 gestational weeks)	–	(67)
–	–	(68)
**Gestational age**		
DMP, DEP, DPP, BMPP, DNHP, BBP, DNOP, DCHP, DMEP, DBP, DIBP, DBEP, DEHP, and DNP with gestational age	DMP, DEP, DPP, BMPP, DNHP, BBP, DNOP, DCHP, DMEP, DBP, DIBP, DBEP, DEHP, and DNP with preterm birth	(69)
MEHP with gestational age		(70)
MEP with pregnancy duration		(71)
MBzP, MCPP, MEP, and the sum of DEHP metabolites or DBP metabolites with gestational age		(72)
MECPP with gestational length	MEHP, MECPP, and the sum of DEHP metabolites with preterm birth	(73)
	MEHP, MECPP and DEHP with preterm birth	(74)
	MBzP, MBP, MECPP, and the sum of DEHP metabolites and spontaneous preterm birth	(75)
MBP, MiBP with gestational ages	MBP, MiBP, and MHiBP with preterm birth	(76)
	The sum of DEHP metabolites with preterm birth	(77)
	MBP and MEHP with preterm birth	(78)
MEHHP with gestation age		(79)
MBzP with gestational length in female	MBzP with gestational length in male	(80)
MMP, MOP and MNP with gestation age	MEHP with gestation age	(81)
MCPP with gestational ages	MEHHP with gestational ages	(82)
	LMWP metabolites with gestational ages	(83)
MEHP and MEOHP with preterm birth	MEHP and MEOHP with gestational ages	(84)
–	–	(85)
–	–	(86)

A Danish prospective cohort study (*n* = 128) reported that maternal urinary MEHP concentrations measured on the 10th day of the last menstrual period before conception were positively associated with subclinical embryonic loss (<6 weeks' gestation), but inversely associated with clinical pregnancy loss (>6 weeks of gestation) (59). However, in a large population study (*n* = 3,220), maternal urinary concentrations of MEP, MEOHP, MEHHP, and ΣHMWP were significantly correlated to higher odds of embryonic loss (during 6–10 weeks' gestation), and MEHHP was positively associated with fetal loss (during 11–27 gestational weeks) after stratified analysis by gestational weeks (60). With respect to subfertile couples conceiving through medically assisted reproduction, urinary concentrations of DEHP metabolites (MEHP, MEHHP, MEOHP, and the molar sum of DEHP metabolites) during fertility treatment cycle were positively associated with both subclinical embryonic loss and pregnancy loss before 20 gestational weeks (63). Therefore, all results indicated that embryos were susceptible to phthalates, and the smaller gestational age, the more sensitive to phthalate exposure.

Several case-control studies demonstrated associations that higher levels of certain phthalate metabolites in urine were correlated to an increased odds of missed miscarriage (<20 weeks' gestation) (61), clinical pregnancy loss (62), and unexplained recurrent spontaneous abortion (65). The urinary MMP and MEHP levels were associated with a missed miscarriage, which showed a strong dose–response (61). And the urinary concentrations of MiBP and MBP or the sum of MiBP and MnBP were remarkably higher in the recurrent pregnancy loss patients (64, 65). Furthermore, the logistic analysis revealed that DEHP exposure measured by hair was an important factor contributing to the missed abortion in a Chinese study (66). Taken together, these findings revealed that the exposure of phthalates during early pregnancy led to an increased risk of pregnancy loss and might be an independent risk factor for pregnancy loss.

A study done by Jukic et al. (67), on the contrary, found none of phthalate metabolites had no bearing on the risk of early pregnancy loss (<6 weeks of the last menstrual period), except for the associations between MEOHP and reduced odds of early pregnancy loss. And the phthalate metabolites were not associated with early pregnancy loss in a study with participants who received *in vitro* fertilization or intracytoplasmic sperm injection treatment (81). But, the non-tendency in the odds of early loss may due to the earlier loss being not detected. Hence, more powerful studies with a single standard of pregnancy loss were essential for elucidating the associations between phthalate exposure and pregnancy loss, which could give some sensible advice to the obstetrician.

## Prenatal Phthalate Exposure and Gestational Age

In view of the ubiquitous usage of phthalates in daily life, potential effect on the gestational age for exposing to phthalates during pregnancy had been greatly considered but without consistent conclusions including decreased gestational age (68, 69, 71–73, 76, 80, 82, 84, 85), and increased (70, 72, 79, 87) or no change (83, 86). Preterm birth, completing delivery before 37 weeks of gestation, is associated with a variety of contributing causes and risk factors. Several studies had investigated the effect of phthalate exposure during pregnancy on preterm birth, whereas the results had been suggestive but not fully conclusive (Table 3).

A study focused on Chinese women revealed that 15 phthalate metabolites (dimethyl-phthalate [DMP], diethyl-phthalate [DEP], dipentyl phthalate [DPP], bis[4-methyl-2-pentyl]phthalate, dihexyl phthalate [DNHP], benzyl butyl phthalate [BBP], DnOP, dicylohexyl phthalate [DCHP], bis[2-methoxyethyl] phthalate [DMEP], DBP, di-iso-butyl-phthalate [DiBP], bis[2-n-butoxyethyl] phthalate [DBEP], DEHP, and dinonyl phthalate [DNP]) levels of cord blood were correlated to a shorter gestational age and an increase in preterm delivery (82). While in an Italian study, and association was found between MEHP and the shorter mean gestational age (8.4 days) alone (68). Aside from measuring phthalate metabolites in cord blood, most researches (69, 84, 85) focused on assessing phthalate exposure via maternal urine. After adjustment for possible confounders, a negative association between the pregnancy duration and maternal urinary MEP in the third trimester was observed in a prospective cohort from Poland (*n* = 165), which was of borderline significance (84). A similar result was shown in the cohort study from Michigan (*n* = 68); phthalate metabolites (MBzP, MCPP, MEP ∑DEHPm, and ∑DBPm) measured during the first trimester were related to shorter pregnancy duration, despite being not statistically significant (85). Boss et al. (69) reported that pregnant women had a higher incidence of preterm birth whose urinary concentrations of MEHP, MECPP, and summed DEHP metabolites were higher, whereas MECPP was the only metabolite that showed a shorter gestational length. A prospective observational cohort study first reported that the average maternal urinary level of DEHP metabolites (MEHP, MECPP, and ΣDEHPm) across the duration of pregnancy was positively related to preterm birth being clearly dose-dependent (74). Subsequently, they observed that spontaneous preterm birth was related to levels of MBzP, MBP, MECPP, and the sum of DEHP metabolites at the third trimester and the conclusions were consistent after utilizing nine statistical methods (75, 88). Moreover, the third trimester of pregnancy was the most sensitive period for phthalate exposure on preterm birth (75). Recently, they found the MBP and metabolites of DiBP (MiBP and MHiBP) were related to shorter gestation and more preterm birth, which were stronger at the second study visit (median = 23 gestational weeks) (71). While in another birth cohort done by Ferguson, the urinary summed DEHP metabolites during the third trimester were associated with preterm birth (77). In addition, significant positive associations were reported that preterm birth with maternal urinary MBP and MEHP concentrations during the third trimester after correction by specific gravity or creatinine (78). All findings supported the results that phthalate exposure was related to a short pregnancy duration and preterm birth.

Besides, the effects of maternal phthalate exposure on gestational age differed according to race/ethnicity or fetal gender (72, 73, 76). A longitudinal cohort study found the adverse effect of prenatal DEHP metabolite exposure during the third-trimester on gestational duration, which was somewhat stronger among African American subjects than among Dominican subjects (73). However, Shoaff et al. (72) found that the effect of phthalate exposure and gestational duration was modified by infant sex, not by maternal race (72). Higher MBzP concentrations in maternal urine were negatively associated with gestational length in female infants but positively with gestational duration in male infants (72). And MEHHP concentrations in maternal urine were related to a significant decrease (4.2 days) in gestation age after adjusting for race, which was observed only in males after stratification by gender (76). Taken together, even though these associations varied by infant sex and exposure to different phthalate metabolites, these studies suggest prenatal phthalate exposure led to a shorter gestational age, which could entail morbid consequences for the neonates.

To explore the mechanism for this relationship, Ferguson et al. (58) measured the concentrations of 8-isoprostane in urine, an oxidative stress biomarker, in the same sample and applied for four counterfactual meditation methods. A positive correlation was found between 8-isoprostane levels and preterm, particularly spontaneous preterm birth, which provided causal evidence for the effects of phthalate exposure on preterm birth partly attributing to phthalate-induced oxidative stress (58).

Interestingly, Smarr et al. (80) observed that maternal phthalate (MMP, MnOP, and MiNP) exposures were associated with the shorter gestation duration except for MEHP, but paternal phthalate exposure was in relation to longer gestation duration. Similar conclusions were reported by a recent study; higher urinary MEHHP concentrations during the preimplantation were associated with longer gestation, but MCPP concentrations during the postimplantation increased the odds of shorter gestation (87). Additionally, two studies from America noted that phthalate metabolite concentrations in urine were associated with increased gestational ages (70, 79) and inversely associated with preterm birth (70). But it is difficult to evaluate the clinical significance of the increase in gestational length either only 0.97 days' gestational age per ln-LMWP metabolites increase (79) or ~2 days longer with each tertile increase in urinary MEHP and MEOHP concentrations (70). Besides, the association failed to be detected between gestational length or preterm birth, and phthalate metabolite exposure during pregnancy, but the phthalate esters exposure was assessed by questionnaire or low dose (83, 86).

Besides, the relationships between prenatal phthalate exposure and gestational length or preterm labor were varied by infant sex, the timing of exposure, types of phthalate exposure, race, and country. Given the large sample size and the more scientific method to measure cumulative phthalate exposure in the study done by Ferguson et al. (58, 74, 75), the conclusions that phthalate exposure during pregnancy would increase the odds of preterm delivering seem sufficiently reliable. Hence, steps should be taken to prevent or reduce phthalate exposure during pregnancy, and further studies should be performed to determine which specific phthalate metabolites are most relevant to the risk of preterm birth.

## Prenatal Phthalate Exposure and Growth of Fetal and Child

There were substantial evidence that prenatal phthalate exposure results in abnormal fetal development and adverse perinatal outcome, even persisted in childhood. Those studies, examining the phthalate metabolites in maternal urine samples, maternal meconium or cord blood, reported inverse (82, 84, 85, 89–98), null (68, 72, 79, 83, 99), and positive (85, 97) associations between women exposed to phthalates and its fetal growth. Moreover, a few longitudinal studies (97, 100–107) had assessed associations of certain phthalate exposure during pregnancy and BMI, waist circumference, and risk of overweight/obesity in childhood, and the findings were inconsistent and often sex-specific (Table 4).

**Table 4:** The effect of prenatal phthalate exposure on growth of fetal and child.

**Negative association**	**Positive association**	**References**
**Fetal growth**		
DEHP and MEHP with birth length DBP with birth weight MEHP with birth weight		(89)
MEHP, MEOHP, MEHHP, MBP, and MMP with birth weight		(90)
BBzP with birth weight length and chest circumference DEHP with birth weight, length and head circumference		(91)
MoiNP with neonatal head circumference	MHiNP and the sum of MHiNP and MOiNP with child length	(71)
HMWP metabolites (MECPP, MEHHP, MEOHP) with estimated fetal weight, biparietal diameter, and head circumference	MCNP, MCPP and fetal femoral length MCNP and length	(97)
MEHHP, MEOHP with birth weight in males MEHP, MEHHP, the sum of DEHP with birth length MMP with birth length		(92)
DMEP with birth weight DEP, DNHP, BBP and DNP with abdominal circumference DBP, DCHP, DEHP and DEP with birth length DPP and DBEP with birth length		(69)
ΣDBP (MnBP and MiBP) with birth weight in female	ΣDBP (MnBP and MiBP) with birth weight in males MCPP with birth length in males ΣDBP (MnBP and MiBP) and MCPP with birth length in females	(72)
MMP and MEP with birth weight in females MEHP, MEHHP and MEOHP with birth weight in males	MBP and MEHP with birth weight in males	(93)
Occupational phthalates with fetal weight growth rates or fetal length		(94)
MECPP and DEHP metabolites with head or abdominal circumferences, FL, and estimated or actual fetal weight		(95)
MnBP with head circumference	MBzP with fetal length or birth weight in boys	(96)
MMP, MEP, MiBP, and MnBP and birth heights, head circumferences		(98)
–	–	(85)
–	–	(99)
–	–	(70)
MBP with birth weight in males	MCOP with birth weight in males	(100)
	DEHP metabolites with birth weight in females	
**Child's growth**		
	MEP with weight growth velocity orBMI	(97)
	MEP, MBzP, and ∑DEHPm (MEHP, MEHHP, MEOHP, and MECCP) with overweight or obesity	(101)
	MEP, MBP, MiBP, MBzP, and the sum of DEHP metabolites with childhood BMI, waist circumference or percent body fat	
Non-DEHP metabolites with childhood BMI z-scores, waist circumference or fat mass		(102)
–	–	(103)
–	–	(104)
Phthalate with overweight		(105)
MEP and DEHP with BMI z-scores in girls	MCPP with overweight/obese status MnBP with BMI z-scores in girls	(106)
∑HMWPm with BMI z-scores in boys	∑HMWPm with BMI z-scores in girls	(53)
MiBP, MBzP, MEHP and MECPP with BMI trajectories in males	MECPP with BMI trajectories in female	(107)

### Prenatal Phthalate Exposure and Fetal Growth

Prenatal phthalate exposure restricts the fetal intrauterine growth through down-regulating of placental THR signaling and inhibiting THR-mediated placental vascular endothelial growth factor, placenta growth factor, and insulinlike growth factor expression in mice (108). Most epidemiological studies in humans found that exposure to LMWP metabolites, such as MEP, MBP, MiBP, and HMWP metabolites, MEHHP, and MEOHP, were negatively related to birth weight (82, 85, 89–96), birth length (89, 91, 95), and circumferences of head, chest or abdominal (84, 85, 91, 94, 96, 98).

In nested case-control studies, Zhang et al. found that *in utero* phthalate metabolite exposure (DBP in cord blood and MEHP in meconium) and maternal urinary phthalate levels (such as MMP, MBP, MEHP, MEOHP, and MEHHP) were negatively correlated with birth weight (89, 90). Besides, the concentrations of DEHP in cord blood and MEHP in cord blood or meconium were related to shorter birth length (89). Gao et al. (91) detected modest but significant associations between the cumulative butyl benzyl phthalate (BBzP) and DEHP exposure and impaired fetal growth, such as birth weight, birth length, and chest and head circumference (HC). However, one study showed a positive effect of mono(hydroxyisononyl)-phthalate (MHiNP) and MOiNP on the neonatal length and an adverse association between MOiNP and neonatal HC in the multivariable-adjusted model (84).

Maternal urinary concentrations of HMWP metabolites (MECPP, MEHHP, and MEOHP) were negatively correlated with estimated fetal weight, biparietal diameter, and HC assessed (97). Besides, Zhao et al. (92) found that maternal urinary MEHHP and MEOHP concentrations in the third trimester were negatively related to fetal birth weight; the inverse relation was shown in males, not in females. Besides, they reported that maternal concentrations of MEHP, MEHHP, and SumDEHP were negatively associated with birth length among male infants, but there was a negative association between MMP and birth length in females (92).

The inverse associations between phthalate exposure measured by cord blood and fetal growth were also reported. After adjusting for gestational age, DMEP was associated with low birth weight; DEP, DNHP, BBP, and DNP with a decreased abdominal circumference; DBP, DCHP, DEHP, and DEP with a decreased femur length (FL) among female infants, whereas in male infants, only DPP and DBEP were related to shorter birth length (82). Moreover, Watkins et al. (85) observed maternal urinary ΣDBP (MnBP and MiBP) levels were associated with a decreased birth weight in females but associated with an increase in birth weight among males. Similarly, maternal MMP and MEP exposures were negatively associated with birth weight only among female infants, whereas the positive effect of MBP and MEHP on neonatal birth weight was observed only in males (93). And the adverse effect of MEHP, MEHHP, and MEOHP exposure on birth weight was stronger in male infants with low birth weight (93). Taken together, these findings suggest that exposure to certain phthalates impaired fetal growth parameters, which was modulated by fetal gender (82, 85, 92, 93).

Several studies further investigated the impairment of maternal phthalate exposure on fetal growth, which was estimated in combination with repeated ultrasound measures (94–96). One prospective cohort study pointed out that maternal occupational phthalate exposure adversely influenced fetal weight growth rates and fetal length (94). Ferguson et al. (95) observed that exposure to MECPP and DEHP metabolites during pregnancy would impair fetal growth, like the circumferences of head and abdominal, FL, and estimated or actual fetal weight. Combining repeat measures of exposure biomarker and ultrasound, Casas et al. (96) observed that prenatal MBzP exposure was positively related to FL growth from 20 to 34 gestational weeks and birth weight in boys, whereas MnBP was negatively correlated with HC growth from 12 to 20 gestational weeks (96). And they, however, did fail to find the relationships between DEHP and its metabolite exposure during pregnancy and fetal growth or birth outcomes. Tsai et al. (98) found maternal phthalate (MMP, MEP, MiBP, and MnBP) exposure during the first trimester were unrelated to fetal growth, but exposure during the second or third trimester would affect fetal birth height and HC. Besides, several epidemiological studies also failed to find associations of phthalate metabolite exposure during pregnancy with any of birth outcomes among their newborns from Japan (83), France (99), and Italy (68). Neither HMWP metabolites nor DEHP metabolites were significantly related to any birth outcome by adjustment for potential confounders (72, 79).

A few studies found a positive effect of prenatal phthalate exposure on infant growth outcomes (85, 97, 100). The concentrations of MCiNP and MCPP were positively related to the estimated femoral length, but only MCiNP was positively associated with the actual birth length (97). Also, maternal urinary ΣDBP and MCPP levels before delivery were associated with higher birth weight and birth length among males, respectively, whereas both of them were associated with an increased birth length among females (85). Likewise, the study done by Sathyanarayana et al. (100) observed sex-specific effects of prenatal phthalate exposure on birth weight among term and preterm infants. Regarding term infants, pregnant women exposed to MCiOP was associated with an increased neonatal birth weight only in male infants. And for preterm infants, DEHP metabolite exposure was related to higher birth weight among females, but MBP exposure was negatively correlated with birth weight in male infants (100).

In conclusion, these results indicated the generally negative effects of pregnant women exposed to phthalate during pregnancy on fetal or neonatal growth, which was varied by infant sex, prematurity, and timing of phthalate exposure.

### Prenatal Phthalate Exposure and Child's Growth

Contrary to the negative effect for fetal growth, maternal urinary MEP concentrations were positively associated with the weight growth velocity of offspring from 2 to 5 years and BMI at 5 years of age (97). In another study extending the follow-up time to 12 years and measuring more than 11 phthalate metabolites in maternal urine twice during pregnancy, prenatal MEP, MBzP, and ∑DEHPm (MEHP, MEHHP, MEOHP, and MECPP) level showed a positive association with risk of overweight and obesity from 5 to 12 years of age (101). Additionally, the levels of MEP, MBP, MiBP, MBzP, and ∑DEHPm were also positively correlated with BMI, waist circumference, and percent body fat of offspring, which, however, weakened with increasing age (101).

On the contrary, other studies reported inverse or null associations between prenatal phthalate exposure and child's growth (53, 102–107). One prospective study showed that maternal urinary concentrations of non-DEHP metabolites before delivery, but not DEHP metabolites, were related to a decrease in fat mass, waist circumference, and BMI *Z* scores of boys at 5 and 7 years ages (102). Similarly, two studies from America failed to detected associations between prenatal phthalate exposure and child adiposity at 1–8 years of age (103) or body fat from 4 to 9 years of age (104). After multipollutant analysis, a Spanish study reported that prenatal phthalate exposure might decrease overweight in the child at 7 years (105). In a pooled study including 707 American children with three cohorts, the relationships of phthalate exposure and offspring growth at ages 4–7 years were inconsistent. Buckley et al. (106) indicated that concentrations of DEHP and MEP in maternal urine were associated with decreased BMI *Z* scores in girls, whereas the MnBP was related to higher BMI *Z* scores in girls (106). Moreover, prenatal MCPP exposure would increase the incidence of overweight/obesity in both boys and girls.

Similarly, a prospective Spanish cohort study reported sex-specific associations between maternal phthalate exposure and childhood anthropometry as well. Maternal urinary concentrations of ∑HMWPm (MBzP, MEHP MEHHP, MEOHP, and MECPP) were inversely related to BMI *Z* score in boys at each visit time (at 1, 4, and 7 years), which would weaken with the child age increasing, but positively associated with BMI *Z* score in girls (53). Yang et al. (107) reported the different influences of maternal phthalate exposure on BMI trajectories from birth to 14 years among boys and girls (107). The highest tertile of MECPP exposure among girls had the highest BMI trajectory, but low tertile exposure of MiBP, MBzP, MEHP, and MECPP predicted the highest BMI trajectory during adolescence of male children (107).

Therefore, these discrepant conclusions may be the result of the various types or timing of phthalate measured and the different follow-up time. Prenatal phthalate exposures had sexually dimorphic effects on physical development. Further studies are needed to elucidate the effect of prenatal phthalate exposures on postnatal growth since adolescence to adult and provide further guidelines for an effective regulatory policy.

## Prenatal Phthalate Exposure and Genital Abnormalities

The immature reproductive tract is susceptible to the effects of phthalates, such as the impairment of fetal masculinization and genital development. With increasing concern, investigators hypothesized that a syndrome of reproductive abnormalities may be induced by intrauterine phthalate exposure, which includes shortened anogenital distance (AGD), hypospadias, cryptorchidism, and so on (109). Dysfunction of Leydig cells, disturbance of sex hormone levels and reduction of insulin-like hormone 3 expression may be the potential mechanisms for the genital abnormalities in newborns or adolescent (110) (Table 5).

**Table 5:** The effect of prenatal phthalate exposure on genital abnormalities.

**Negative association**	**Positive association**	**References**
**Anogenital distance**		
–	–	(110)
MBP with AGI in females	–	(111)
MEP, MEP, MBP, MBzP, and MiBP with AGI in male infants		(112)
MEHP, MEOHP, and MEHHP with ADG in male		(113)
MEHP with AGI in male		(114)
MEHP with AGD		(115)
DiNP metabolites (oh-MMeOP and oxo-MMeOP) and ∑DiNPm with AGD		(116)
MEHP with anopenile distance in boy MEHP with anoclitoral distance and anofourchette distance in girls	The sum of MiBP and MBP with anoscrotal distance in boy	(117)
MBzP with anoclitoris distance	MEP with anoclitoris distance MnBP and ∑LMWPm (MMP, MEP, MnBP, MCHP, MCPP, and MBzP) with anopenile distance in males	(118)
**Cryptorchidism**		
	DEHP metabolites with incomplete testicular descent	(119)
	Phthalate with cryptorchidism	(120)
–	–	(121)
–	–	(122)
–	–	(33)
–	–	(37)
**Hypospadias**		
	Occupation phthalate with hypospadias	(123)
	Occupation phthalate with hypospadias	(124)
	Occupation phthalate with hypospadias	(125)
Occupation phthalate with hypospadias		(121)
–	–	(33)

### Prenatal Phthalate Exposure and Anogenital Measurements

Anogenital distance, distance from anus to the genitalia, is a sexually dimorphic trait as a marker of prenatal androgen exposure in humans. Several studies had investigated whether prenatal phthalate exposures will alter AGD among offsprings in humans, and the results were less consistent (111–118, 126, 127).

Two studies did not find inverse associations between maternal phthalate exposure and AGD in males (112, 126). One study from Denmark (*n* = 245) showed the lower phthalate levels measured at ~28 gestational weeks, and no significant association was discovered between any phthalate exposure during pregnancy and AGD or penile width of offspring (126). Another one also found no statistical association between the maternal urinary phthalate metabolite concentrations (including DEHP) and male newborns' anogenital index (AGI = AGD/body weight) (*n* = 33), whereas the higher concentrations of MBP in amniotic fluid were negatively associated with the AGI in females at birth (112).

Swan et al. (113) found that prenatal phthalate exposure was inversely associated with AGD and AGI in male infants of 2–36 months of age. Moreover, they found a different conclusion in a later publication that exposure to DEHP metabolites (MEHP, MEOHP, and MEHHP) during the first trimester, which is the critical period of genital development, was correlated with shorter AGD in males (114). However, the associations failed to be found in female newborns (113, 114). Taken together, these results suggest that maternal phthalate exposure at environmental levels during pregnancy, although was of low levels, would adversely affect genital development.

Similar findings were reported that prenatal exposure to DEHP metabolites was significantly inversely related to the AGI of male newborns (115) or the AGD within 24–48 h of birth (116). And a Swedish study reported the association between DiNP metabolites (oh-MmeOP, oxo-MmeOP, and ∑DiNPm) and shorter boys' AGD (117). In summary, these studies demonstrated inverse associations of prenatal phthalate exposures and AGD for neonatal boys, but with no association in girls (113–117).

In a prospective study including 187 African American and 193 white mothers, Wenzel et al. (118) investigated the impact of race or fetal sex on associations between prenatal exposure to phthalate and fetal genital development. A significant association for higher MEHP exposure during the second trimester of gestation and shorter anopenile distance of boys and the positive relationship of the ∑DBPm (MiBP and MBP) with anoscrotal distance in boys was stronger for whites than for African Americans (118). In newborn girls, they detected inverse associations for MEHP with anoclitoral distance and anofourchette distance (118). It is clear that the influence of prenatal phthalate exposure on anogenital measurements was varied by race and sex, which was important for future health interventions.

Recently, Arbuckle et al. (127) found the anoclitoris distance in females was positively related to MEP concentration in maternal urine but negatively to MBzP, and no phthalate metabolite exposure was associated with anofourchette distances. But for male infants, urinary concentrations of MnBP and ∑LMWPm (MMP, MEP, MnBP, mono cyclohexyl phthalate [MCHP], MCPP, and MBzP) were related to longer anopenile distance, and none of the phthalate metabolites were correlated with anoscrotal distance (127). In their later study, moreover, they found that stressful life events during pregnancy would impact the effect of phthalate on AGD (111). To summarize, although available scientific evidence was limited, it needs more concern because of the possible adverse effects on genital development.

### Prenatal Phthalate Exposure and Cryptorchidism

Cryptorchidism is the most common congenital malformation in newborn boys, which is a potential cause of infertility and testicular cancer. Swan (119) reported that higher urinary DEHP metabolite levels were significantly related to a higher incidence of incomplete testicular descent. And the evidence from a large sample prospective study (6,246 boys and 102 cryptorchid cases) suggests that maternal phthalate exposure was a risk factor for cryptorchidism at birth, whereas the phthalate exposure was assessed only by semiquantitative questions (120).

However, similar associations failed to be found between cryptorchidism and maternal phthalate exposure, which was measured not only in maternal spot urine sample (121) but also in cord blood (122). Jensen et al. (33) did not observe the associations between cryptorchidism and the amniotic fluid levels of MECPP and mono(4-methyl-7-carboxyheptyl) phthalate (7cx-MmeHP) in the second trimester. Furthermore, a cohort study following boys to 20 years of age (*n* = 216) found only two boys had cryptorchidism, and no apparent effect of phthalate exposure was found (37).

Therefore, there was no clear-cut relationship between *in utero* phthalate exposure and cryptorchidism, which was needed to further research.

### Prenatal Phthalate Exposure and Hypospadias

Hypospadias is a congenital abnormality of the male genitalia characterized by incomplete development of the urethra, in which the external urethral opening is abnormal in position. Reported rises in the prevalence of hypospadias affecting male infants may be a result of exposure to phthalate with estrogenic or antiandrogenic properties. These studies mostly focused on the effect of occupational phthalate exposure on hypospadias (123–125).

In a study with 471 cases and 490 controls, Ormond et al. (123) indicated a high rate of hypospadias in children whose mothers were exposed to phthalate (especially exposed to hairspray) during the first trimester of pregnancy. Consistent with this result, a team from Britain showed that the children whose mother was a hairdresser had an increased risk of hypospadias as a result of the occupational phthalate exposure during pregnancy (124). However, the results should be treated with caution, because this correlation was observed only in 1992–1996, but not confirmed between 1980 and 1989 (124). This discrepancy was possibly due to the difficulty of separating the effects of occupation and social class as well as other potential confounders.

A large study (1,202 cases and 2,583 controls) supported the same conclusion that children whose mothers working in the hairdressing, beauty, or cleaning industry had an increased risk of hypospadias (125). However, Chevrier et al. (121) observed a negative trend toward hypospadias and maternal occupational phthalate exposure, but it was lacking power owing to the small sample size (only 21 hypospadias cases and 50 controls). Besides, a recent study reported that the levels of phthalate metabolites (MEPP or 7cx-MmeHP) in amniotic fluid collecting at ~16 gestational weeks were not associated with hypospadias, whereas the samples were from the women with a risk of severe malformations or Down syndrome (33).

Human studies showed the inverse associations between parental occupational exposure to phthalates and the hypospadias in infants, while it remains controversial. Thus, it still needed more powerful evidence to confirm the effect of prenatal phthalate exposure on the hypospadias.

## Conclusions

In conclusion, phthalates are a family of ubiquitous synthetic endocrine-disrupting chemicals. As a result of prenatal exposure to phthalate with estrogenic or antiandrogenic properties, growing evidence suggests gonadal hormones and THs of mother and infant had been changed, and circulating levels of total 25(OH)D in pregnant women had been disrupted, which resulted in serious fertility and perinatal complications, and adverse maternal and neonatal outcomes such as preeclampsia, glucose disorders, preterm birth, cryptorchidism, hypospadias, and shorter AGD, in particular, for male newborns, growth retardation in early adolescence and childhood. The relation of prenatal phthalate exposures with maternal and neonatal outcomes in human beings was often sex-specific associations. Because of different findings across the studies described in this article, more conclusive epidemiologic evidence and mechanistic studies are urgently needed. Following these findings, steps should be taken to prevent or reduce phthalate exposure during pregnancy.

## Author Contributions

YQ prepared the initial draft, and critically reviewed the manuscript. WH and XY participated in data collection and organization. HS manuscript revisng. YH interpretation, reviewed the manuscript, and approved submission. All authors contributed to the article and approved the submitted version.

## Conflict of Interest

The authors declare that the research was conducted in the absence of any commercial or financial relationships that could be construed as a potential conflict of interest.

## References

[1] Katsikantami I Sifakis S Tzatzarakis MN Vakonaki E Kalantzi OI Tsatsakis AM. A global assessment of phthalates burden and related links to health effects. Environ Int. (2016) 97:212–36. 10.1016/j.envint.2016.09.013 27669632

[2] Gao D-W Wen Z-D. Phthalate esters in the environment: a critical review of their occurrence, biodegradation, and removal during wastewater treatment processes. Sci Total Environ. (2016) 541:986–1001. 10.1016/j.scitotenv.2015.09.148 26473701

[3] Szewczynska M Pośniak M Dobrzynska E Determination of phthalates in particulate matter and gaseous phase emitted into the air of the working environment. Int J Environ Sci Technol. (2020) 17:175–86. 10.1007/s13762-019-02435-y

[4] Erkekoglu P Gumusel B Environmental effects of endocrine-disrupting chemicals: a special focus on phthalates and bisphenol A. In: Larramendy ML Soloneski S, editors. Environmental Health Risk - Hazardous Factors to Living Species. London: IntechOpen (2016). p. 155–90. 10.5772/62455

[5] Mose T Mortensen GK Hedegaard M Knudsen LE. Phthalate monoesters in perfusate from a dual placenta perfusion system, the placenta tissue and umbilical cord blood. Reprod Toxicol. (2007) 23:83–91. 10.1016/j.reprotox.2006.08.006 17049806

[6] Watkins DJ Sanchez BN Tellez-Rojo MM Lee JM Mercado-Garcia A Blank-Goldenberg C. Phthalate and bisphenol A exposure during *in utero* windows of susceptibility in relation to reproductive hormones and pubertal development in girls. Environ Res. (2017) 159:143–51. 10.1016/j.envres.2017.07.051 28800472 PMC5623649

[7] Barakat R Seymore T Lin PP Park CJ Ko CJ. Prenatal exposure to an environmentally relevant phthalate mixture disrupts testicular steroidogenesis in adult male mice. Environ Res. (2019) 172:194–201. 10.1016/j.envres.2019.02.017 30802670 PMC6511329

[8] Takeuchi S Iida M Kobayashi S Jin K Matsuda T Kojima H. Differential effects of phthalate esters on transcriptional activities via human estrogen receptors alpha and beta, and androgen receptor. Toxicology. (2005) 210:223–33. 10.1016/j.tox.2005.02.002 15840436

[9] Engel A Buhrke T Imber F Jessel S Seidel A Volkel W. Agonistic and antagonistic effects of phthalates and their urinary metabolites on the steroid hormone receptors ERalpha, ERbeta, and AR. Toxicol Lett. (2017) 277:54–63. 10.1016/j.toxlet.2017.05.028 28571686

[10] Jia M Dahlman-Wright K Gustafsson JA. Estrogen receptor alpha and beta in health and disease. Best Pract Res Clin Endocrinol Metab. (2015) 29:557–68. 10.1016/j.beem.2015.04.008 26303083

[11] Han Lu Shen W-J Bittner S Kraemer FB Azhar S. PPARs: regulators of metabolism and as therapeutic targets in cardiovascular disease. Part II: PPAR-β/δ and PPAR-γ. Future Cardiol. (2017) 13:279–96. 10.2217/fca-2017-0019 28581362 PMC5941699

[12] Authority EFS. Opinion of the scientific panel on food additives, flavourings, processing aids and materials in contact with food (AFC) on a request related to a 12th list of substances for food contact materials. EFSA J. (2006) 1:395–401. 10.2903/j.efsa.2006.39532626247

[13] Bhat VS Durham JL Ball GL English JC. Derivation of an oral reference dose (RfD) for the nonphthalate alternative plasticizer 1, 2-cyclohexane dicarboxylic acid, di-isononyl ester (DINCH). J Toxicol Environ Health B Crit Rev. (2014) 17:63–94. 10.1080/10937404.2013.876288 24627975

[14] Engel A Buhrke T Kasper S Behr A-C Braeuning A Jessel S. The urinary metabolites of DINCH have an impact on the activities of the human nuclear receptors ERα, ERβ, AR, PPARα and PPARγ. Toxicol Lett. (2018) 287:83–91. 10.1016/j.toxlet.2018.02.006 29421333

[15] Huang PC Kuo PL Guo YL Liao PC Lee CC. Associations between urinary phthalate monoesters and thyroid hormones in pregnant women. Hum Reprod. (2007) 22:2715–22. 10.1093/humrep/dem205 17704099

[16] Huang PC Tsai CH Liang WY Li SS Huang HB Kuo PL. Early phthalates exposure in pregnant women is associated with alteration of thyroid hormones. PLoS ONE. (2016) 11:e0159398. 10.1371/journal.pone.0159398 27455052 PMC4959782

[17] Yao HY Han Y Gao H Huang K Ge X Xu YY. Maternal phthalate exposure during the first trimester and serum thyroid hormones in pregnant women and their newborns. Chemosphere. (2016) 157:42–8. 10.1016/j.chemosphere.2016.05.023 27208644

[18] Johns LE Ferguson KK Soldin OP Cantonwine DE Rivera-Gonzalez LO Del Toro LV. Urinary phthalate metabolites in relation to maternal serum thyroid and sex hormone levels during pregnancy: a longitudinal analysis. Reprod Biol Endocrinol. (2015) 13:4. 10.1186/1477-7827-13-4 25596636 PMC4326411

[19] Johns LE Ferguson KK McElrath TF Mukherjee B Meeker JD. Associations between repeated measures of maternal urinary phthalate metabolites and thyroid hormone parameters during pregnancy. Environ Health Perspect. (2016) 124:1808–15. 10.1289/EHP170 27152641 PMC5089879

[20] Gao H Wu W Xu Y Jin Z Bao H Zhu P. Effects of prenatal phthalate exposure on thyroid hormone concentrations beginning at the embryonic stage. Sci Rep. (2017) 7:13106. 10.1038/s41598-017-13672-x 29026179 PMC5638801

[21] Huang H-B Kuo P-L Chang J-W Jaakkola JJK Liao K-W Huang P-C. Longitudinal assessment of prenatal phthalate exposure on serum and cord thyroid hormones homeostasis during pregnancy - Tainan birth cohort study (TBCS). Sci Total Environ. (2018) 619–620:1058–65. 10.1016/j.scitotenv.2017.11.048 29734584

[22] Cathey AL Watkins D Rosario ZY Velez C Alshawabkeh AN Cordero JF. Associations of phthalates and phthalate replacements with CRH and other hormones among pregnant women in puerto rico. J Endocr Soc. (2019) 3:1127–49. 10.1210/js.2019-00010 31093596 PMC6510018

[23] Romano ME Eliot MN Zoeller RT Hoofnagle AN Calafat AM Karagas MR. Maternal urinary phthalate metabolites during pregnancy and thyroid hormone concentrations in maternal and cord sera: the HOME Study. Int J Hyg Environ Health. (2018) 221:623–31. 10.1016/j.ijheh.2018.03.010 29606598 PMC5972051

[24] Kuo FC Su SW Wu CF Huang MC Shiea J Chen BH. Relationship of urinary phthalate metabolites with serum thyroid hormones in pregnant women and their newborns: a prospective birth cohort in Taiwan. PLoS ONE. (2015) 10:e0123884. 10.1371/journal.pone.0123884 26042594 PMC4456348

[25] Morgenstern R Whyatt RM Insel BJ Calafat AM Liu X Rauh VA. Phthalates and thyroid function in preschool age children: sex specific associations. Environ Int. (2017) 106:11–8. 10.1016/j.envint.2017.05.007 28554096 PMC5533628

[26] Sathyanarayana S Butts S Wang C Barrett E Nguyen R Schwartz SM. Early prenatal phthalate exposure, sex steroid hormones, and birth outcomes. J Clin Endocrinol Metab. (2017) 102:1870–8. 10.1210/jc.2016-3837 28324030 PMC5470772

[27] Sathyanarayana S Barrett E Butts S Wang C Swan SH. Phthalate exposure and reproductive hormone concentrations in pregnancy. Reproduction. (2014) 147:401–9. 10.1530/REP-13-0415 24196015 PMC3943643

[28] Hart R Doherty DA Frederiksen H Keelan JA Hickey M Sloboda D. The influence of antenatal exposure to phthalates on subsequent female reproductive development in adolescence: a pilot study. Reproduction. (2014) 147:379–90. 10.1530/REP-13-0331 24025997

[29] Lin L-C Wang S-L Chang Y-C Huang P-C Cheng J-T Su P-H. Associations between maternal phthalate exposure and cord sex hormones in human infants. Chemosphere. (2011) 83:1192–9. 10.1016/j.chemosphere.2010.12.079 21272909

[30] Araki A Mitsui T Miyashita C Nakajima T Naito H Ito S. Association between maternal exposure to di(2-ethylhexyl) phthalate and reproductive hormone levels in fetal blood: the hokkaido study on environment and children's health. PLoS ONE. (2014) 9:e109039. 10.1371/journal.pone.0109039 25296284 PMC4189794

[31] Kolatorova L Vitku J Vavrous A Hampl R Adamcova K Simkova M. Phthalate metabolites in maternal and cord plasma and their relations to other selected endocrine disruptors and steroids. Physiol Res. (2018) 67(Suppl. 3):S473–87. 10.33549/physiolres.933962 30484674

[32] Araki A Mitsui T Goudarzi H Nakajima T Miyashita C Itoh S. Prenatal di(2-ethylhexyl) phthalate exposure and disruption of adrenal androgens and glucocorticoids levels in cord blood: the hokkaido study. Sci Total Environ. (2017) 581–582:297–304. 10.1016/j.scitotenv.2016.12.124 28043700

[33] Jensen MS Anand-Ivell R Norgaard-Pedersen B Jonsson BA Bonde JP Hougaard DM. Amniotic fluid phthalate levels and male fetal gonad function. Epidemiology. (2015) 26:91–9. 10.1097/EDE.0000000000000198 25384265

[34] Watkins DJ Tellez-Rojo MM Ferguson KK Lee JM Solano-Gonzalez M Blank-Goldenberg C. *In utero* and peripubertal exposure to phthalates and BPA in relation to female sexual maturation. Environ Res. (2014) 134:233–41. 10.1016/j.envres.2014.08.010 25173057 PMC4262586

[35] Ferguson KK Peterson KE Lee JM Mercado-Garcia A Blank-Goldenberg C Tellez-Rojo MM. Prenatal and peripubertal phthalates and bisphenol A in relation to sex hormones and puberty in boys. Reprod Toxicol. (2014) 47:70–6. 10.1016/j.reprotox.2014.06.002 24945889 PMC4117729

[36] Watkins DJ Sánchez BN Téllez-Rojo MM Lee JM Mercado-García A Blank-Goldenberg C. Impact of phthalate and BPA exposure during *in utero* windows of susceptibility on reproductive hormones and sexual maturation in peripubertal males. Environ Health. (2017) 16:69. 10.1186/s12940-017-0278-5 28637469 PMC5480112

[37] Hart RJ Frederiksen H Doherty DA Keelan JA Skakkebaek NE Minaee NS. The possible impact of antenatal exposure to ubiquitous phthalates upon male reproductive function at 20 years of age. Front Endocrinol. (2018) 9:288. 10.3389/fendo.2018.00288 29922230 PMC5996240

[38] Johns LE Ferguson KK Cantonwine DE McElrath TF Mukherjee B Meeker JD. Urinary BPA and phthalate metabolite concentrations and plasma vitamin D levels in pregnant women: a repeated measures analysis. Environ Health Perspect. (2017) 125:087026. 10.1289/EHP1178 28934718 PMC5783673

[39] Springer D Jiskra J Limanova Z Zima T Potlukova E. Thyroid in pregnancy: from physiology to screening. Crit Rev Clin Lab Sci. (2017) 54:102–16. 10.1080/10408363.2016.1269309 28102101

[40] Bodnar LM Platt RW Simhan HN. Early-pregnancy vitamin D deficiency and risk of preterm birth subtypes. Obstet Gynecol. (2015) 125:439–47. 10.1097/AOG.0000000000000621 25569002 PMC4304969

[41] Murthi P Yong HE Ngyuen TP Ellery S Singh H Rahman R. Role of the placental vitamin D receptor in modulating feto-placental growth in fetal growth restriction and preeclampsia-affected pregnancies. Front Physiol. (2016) 7:43. 10.3389/fphys.2016.00043 26924988 PMC4757640

[42] Robledo CA Peck JD Stoner J Calafat AM Carabin H Cowan L. Urinary phthalate metabolite concentrations and blood glucose levels during pregnancy. Int J Hyg Environ Health. (2015) 218:324–30. 10.1016/j.ijheh.2015.01.005 25726127 PMC4724204

[43] Fisher BG Frederiksen H Andersson AM Juul A Thankamony A Ong KK. Serum phthalate and triclosan levels have opposing associations with risk factors for gestational diabetes mellitus. Front Endocrinol. (2018) 9:99. 10.3389/fendo.2018.00099 29593656 PMC5859030

[44] Shaffer RM Ferguson KK Sheppard L James-Todd T Butts S Chandrasekaran S. Maternal urinary phthalate metabolites in relation to gestational diabetes and glucose intolerance during pregnancy. Environ Int. (2019) 123:588–96. 10.1016/j.envint.2018.12.021 30622083 PMC6347428

[45] Shapiro GD Dodds L Arbuckle TE Ashley-Martin J Fraser W Fisher M. Exposure to phthalates, bisphenol A and metals in pregnancy and the association with impaired glucose tolerance and gestational diabetes mellitus: the MIREC study. Environ Int. (2015) 83:63–71. 10.1016/j.envint.2015.05.016 26101084

[46] James-Todd TM Meeker JD Huang T Hauser R Ferguson KK Rich-Edwards JW. Pregnancy urinary phthalate metabolite concentrations and gestational diabetes risk factors. Environ Int. (2016) 96:118–26. 10.1016/j.envint.2016.09.009 27649471 PMC5304919

[47] Redman C Sargent I. Latest advances in understanding preeclampsia. Science. (2005) 308:1592–4. 10.1126/science.1111726 15947178

[48] Han X Li J Wang Y Xu S Li Y Liu H. Association between phthalate exposure and blood pressure during pregnancy. Ecotoxicol Environ Saf. (2019) 189:109944. 10.1016/j.ecoenv.2019.109944 31757513

[49] Cantonwine D Meeker J Ferguson K Mukherjee B Hauser R McElrath T. Urinary concentrations of bisphenol a and phthalate metabolites measured during pregnancy and risk of preeclampsia. Environ Health Perspect. (2016) 124:1651–5. 10.1289/EHP188 27177253 PMC5047771

[50] Werner EF Braun JM Yolton K Khoury JC Lanphear BP. The association between maternal urinary phthalate concentrations and blood pressure in pregnancy: the HOME study. Environ Health. (2015) 14:75. 10.1186/s12940-015-0062-3 26380974 PMC4574131

[51] Philips EM Trasande L Kahn LG Gaillard R Steegers EAP Jaddoe VWV. Early pregnancy bisphenol and phthalate metabolite levels, maternal hemodynamics and gestational hypertensive disorders. Hum Reprod. (2019) 34:365–73. 10.1093/humrep/dey364 30576447 PMC6343467

[52] Warembourg C Basagana X Seminati C de Bont J Granum B Lyon-Caen S. Exposure to phthalate metabolites, phenols and organophosphate pesticide metabolites and blood pressure during pregnancy. Int J Hyg Environ Health. (2019) 222:446–54. 10.1016/j.ijheh.2018.12.011 30595366

[53] Valvi D Casas M Romaguera D Monfort N Ventura R Martinez D. Prenatal phthalate exposure and childhood growth and blood pressure: evidence from the Spanish INMA-sabadell birth cohort study. Environ Health Perspect. (2015) 123:1022–9. 10.1289/ehp.1408887 25850106 PMC4590754

[54] Warembourg C Maitre L Tamayo-Uria I Fossati S Roumeliotaki T Aasvang GM. Early-life environmental exposures and blood pressure in children. J Am Coll Cardiol. (2019) 74:1317–28. 10.1016/j.jacc.2019.06.069 31488269 PMC8713646

[55] Lin Y Wei J Li Y Chen J Zhou Z Song L. Developmental exposure to di(2-ethylhexyl) phthalate impairs endocrine pancreas and leads to long-term adverse effects on glucose homeostasis in the rat. Am J Physiol Endocrinol Metab. (2011) 301:E527–38. 10.1152/ajpendo.00233.2011 21673306

[56] Gingrich J Ticiani E Veiga-Lopez A. Placenta disrupted: endocrine disrupting chemicals and pregnancy. Trends Endocrinol Metab. (2020) 31:508–24. 10.1016/j.tem.2020.03.003 32249015 PMC7395962

[57] Wu F Tian F-J Lin Y. Oxidative stress in placenta: health and diseases. Biomed Res Int. (2015) 2015:293271. 10.1155/2015/293271 26693479 PMC4676991

[58] Ferguson KK Chen YH VanderWeele TJ McElrath TF Meeker JD Mukherjee B. Mediation of the relationship between maternal phthalate exposure and preterm birth by oxidative stress with repeated measurements across pregnancy. Environ Health Perspect. (2017) 125:488–94. 10.1289/EHP282 27352406 PMC5332184

[59] Toft G Jonsson BA Lindh CH Jensen TK Hjollund NH Vested A. Association between pregnancy loss and urinary phthalate levels around the time of conception. Environ Health Perspect. (2012) 120:458–63. 10.1289/ehp.1103552 22113848 PMC3295336

[60] Gao H Zhang YW Huang K Yan SQ Mao LJ Ge X. Urinary concentrations of phthalate metabolites in early pregnancy associated with clinical pregnancy loss in Chinese women. Sci Rep. (2017) 7:6800. 10.1038/s41598-017-06450-2 28754983 PMC5533765

[61] Yi H Gu H Zhou T Chen Y Wang G Jin Y. A pilot study on association between phthalate exposure and missed miscarriage. Eur Rev Med Pharmacol Sci. (2016) 20:1894–902. 27212185

[62] Mu D Gao F Fan Z Shen H Peng H Hu J Levels of phthalate metabolites in urine of pregnant women and risk of clinical pregnancy loss. Environ Sci Technol. (2015) 49:10651–7. 10.1021/acs.est.5b02617 26251123

[63] Messerlian C Wylie BJ Minguez-Alarcon L Williams PL Ford JB Souter IC. Urinary concentrations of phthalate metabolites and pregnancy loss among women conceiving with medically assisted reproduction. Epidemiology. (2016) 27:879–88. 10.1097/EDE.0000000000000525 27299194 PMC5248552

[64] Liao KW Kuo PL Huang HB Chang JW Chiang HC Huang PC. Increased risk of phthalates exposure for recurrent pregnancy loss in reproductive-aged women. Environ Pollut. (2018) 241:969–77. 10.1016/j.envpol.2018.06.022 30029331

[65] Peng F Ji W Zhu F Peng D Yang M Liu R. A study on phthalate metabolites, bisphenol A and nonylphenol in the urine of Chinese women with unexplained recurrent spontaneous abortion. Environ Res. (2016) 150:622–8. 10.1016/j.envres.2016.04.003 27156842

[66] Zhao R Wu Y Zhao F Lv Y Huang D Wei J. The risk of missed abortion associated with the levels of tobacco, heavy metals and phthalate in hair of pregnant woman: a case control study in Chinese women. Medicine (Baltimore). (2017) 96:e9388. 10.1097/MD.0000000000009388 29390543 PMC5758245

[67] Jukic AM Calafat AM McConnaughey DR Longnecker MP Hoppin JA Weinberg CR. Urinary concentrations of phthalate metabolites and bisphenol a and associations with follicular-phase length, luteal-phase length, fecundability, and early pregnancy loss. Environ Health Perspect. (2016) 124:321–8. 10.1289/ehp.1408164 26161573 PMC4786975

[68] Latini G De Felice C Presta G Del Vecchio A Paris I Ruggieri F. *In utero* exposure to di-(2-ethylhexyl)phthalate and duration of human pregnancy. Environ Health Perspect. (2003) 111:1783–5. 10.1289/ehp.6202 14594632 PMC1241724

[69] Boss J Zhai J Aung MT Ferguson KK Johns LE McElrath TF. Associations between mixtures of urinary phthalate metabolites with gestational age at delivery: a time to event analysis using summative phthalate risk scores. Environ Health. (2018) 17:56. 10.1186/s12940-018-0400-3 29925380 PMC6011420

[70] Adibi JJ Hauser R Williams PL Whyatt RM Calafat AM Nelson H. Maternal urinary metabolites of Di-(2-Ethylhexyl) phthalate in relation to the timing of labor in a US multicenter pregnancy cohort study. Am J Epidemiol. (2009) 169:1015–24. 10.1093/aje/kwp001 19251754 PMC2727228

[71] Ferguson KK Rosen EM Rosario Z Feric Z Calafat AM McElrath TF. Environmental phthalate exposure and preterm birth in the PROTECT birth cohort. Environ Int. (2019) 132:105099. 10.1016/j.envint.2019.105099 31430608 PMC6754790

[72] Shoaff JR Romano ME Yolton K Lanphear BP Calafat AM Braun JM. Prenatal phthalate exposure and infant size at birth and gestational duration. Environ Res. (2016) 150:52–8. 10.1016/j.envres.2016.05.033 27236572 PMC5003714

[73] Whyatt RM Adibi JJ Calafat AM Camann DE Rauh V Bhat HK. Prenatal di(2-ethylhexyl)phthalate exposure and length of gestation among an inner-city cohort. Pediatrics. (2009) 124:e1213–20. 10.1542/peds.2009-0325 19948620 PMC3137456

[74] Ferguson KK McElrath TF Meeker JD. Environmental phthalate exposure and preterm birth. JAMA Pediatr. (2014) 168:61–7. 10.1001/jamapediatrics.2013.3699 24247736 PMC4005250

[75] Ferguson KK McElrath TF Ko YA Mukherjee B Meeker JD. Variability in urinary phthalate metabolite levels across pregnancy and sensitive windows of exposure for the risk of preterm birth. Environ Int. (2014) 70:118–24. 10.1016/j.envint.2014.05.016 24934852 PMC4104181

[76] Weinberger B Vetrano AM Archer FE Marcella SW Buckley B Wartenberg D. Effects of maternal exposure to phthalates and bisphenol A during pregnancy on gestational age. J Matern Fetal Neonatal Med. (2014) 27:323–7. 10.3109/14767058.2013.815718 23795657 PMC3996679

[77] Ferguson KK Rosen EM Barrett ES Nguyen RHN Bush N McElrath TF. Joint impact of phthalate exposure and stressful life events in pregnancy on preterm birth. Environ Int. (2019) 133(Pt B):105254. 10.1016/j.envint.2019.105254 31675562 PMC6924167

[78] Meeker JD Hu H Cantonwine DE Lamadrid-Figueroa H Calafat AM Ettinger AS. Urinary phthalate metabolites in relation to preterm birth in Mexico city. Environ Health Perspect. (2009) 117:1587–92. 10.1289/ehp.0800522 20019910 PMC2790514

[79] Wolff MS Engel SM Berkowitz GS Ye X Silva MJ Zhu C. Prenatal phenol and phthalate exposures and birth outcomes. Environ Health Perspect. (2008) 116:1092–7. 10.1289/ehp.11007 18709157 PMC2516577

[80] Smarr MM Grantz KL Sundaram R Maisog JM Kannan K Louis GM. Parental urinary biomarkers of preconception exposure to bisphenol A and phthalates in relation to birth outcomes. Environ Health. (2015) 14:73. 10.1186/s12940-015-0060-5 26362861 PMC4567813

[81] Deng T Du Y Wang Y Teng X Hua X Yuan X. The associations of urinary phthalate metabolites with the intermediate and pregnancy outcomes of women receiving IVF/ICSI treatments: a prospective single-center study. Ecotoxicol Environ Saf. (2020) 188:109884. 10.1016/j.ecoenv.2019.109884 31706241

[82] Huang Y Li J Garcia JM Lin H Wang Y Yan P. Phthalate levels in cord blood are associated with preterm delivery and fetal growth parameters in Chinese women. PLoS ONE. (2014) 9:e87430. 10.1371/journal.pone.0087430 24503621 PMC3913614

[83] Suzuki Y Niwa M Yoshinaga J Mizumoto Y Serizawa S Shiraishi H. Prenatal exposure to phthalate esters and PAHs and birth outcomes. Environ Int. (2010) 36:699–704. 10.1016/j.envint.2010.05.003 20605637

[84] Polanska K Ligocka D Sobala W Hanke W. Effect of environmental phthalate exposure on pregnancy duration and birth outcomes. Int J Occup Med Environ Health. (2016) 29:683–97. 10.13075/ijomeh.1896.00691 27443763

[85] Watkins DJ Milewski S Domino SE Meeker JD Padmanabhan V. Maternal phthalate exposure during early pregnancy and at delivery in relation to gestational age and size at birth: a preliminary analysis. Reprod Toxicol. (2016) 65:59–66. 10.1016/j.reprotox.2016.06.021 27352641 PMC5067196

[86] Burdorf A Brand T Jaddoe VW Hofman A Mackenbach JP Steegers EA. The effects of work-related maternal risk factors on time to pregnancy, preterm birth and birth weight: the generation R study. Occup Environ Med. (2011) 68:197–204. 10.1136/oem.2009.046516 21172792

[87] Chin HB Jukic AM Wilcox AJ Weinberg CR Ferguson KK Calafat AM. Association of urinary concentrations of early pregnancy phthalate metabolites and bisphenol A with length of gestation. Environ Health. (2019) 18:80. 10.1186/s12940-019-0522-2 31470855 PMC6717338

[88] Chen YH Ferguson KK Meeker JD McElrath TF Mukherjee B. Statistical methods for modeling repeated measures of maternal environmental exposure biomarkers during pregnancy in association with preterm birth. Environ Health. (2015) 14:9. 10.1186/1476-069X-14-9 25619201 PMC4417225

[89] Zhang Y Lin L Cao Y Chen B Zheng L Ge R-S. Phthalate levels and low birth weight: a nested case-control study of Chinese newborns. J Pediatr. (2009) 155:500–4. 10.1016/j.jpeds.2009.04.007 19555962 PMC12151091

[90] Song Q Li R Zhao Y Zhu Q Xia B Chen S. Evaluating effects of prenatal exposure to phthalates on neonatal birth weight: structural equation model approaches. Chemosphere. (2018) 205:674–81. 10.1016/j.chemosphere.2018.04.063 29723725

[91] Gao H Xu YY Huang K Ge X Zhang YW Yao HY. Cumulative risk assessment of phthalates associated with birth outcomes in pregnant Chinese women: A prospective cohort study. Environ Pollut. (2017) 222:549–56. 10.1016/j.envpol.2016.11.026 28024814

[92] Zhao Y Chen L Li LX Xie CM Li D Shi HJ. Gender-specific relationship between prenatal exposure to phthalates and intrauterine growth restriction. Pediatr. Res. (2014) 76:401–8. 10.1038/pr.2014.103 25003910

[93] Zhang Y-W Gao H Mao L-J Tao X-Y Ge X Huang K. Effects of the phthalate exposure during three gestation periods on birth weight and their gender differences: a birth cohort study in China. Sci Total Environ. (2018) 613–614:1573–8. 10.1016/j.scitotenv.2017.08.319 28886917

[94] Snijder CA Roeleveld N Te Velde E Steegers EA Raat H Hofman A. Occupational exposure to chemicals and fetal growth: the generation R study. Hum Reprod. (2012) 27:910–20. 10.1093/humrep/der437 22215632 PMC3279127

[95] Ferguson KK Meeker JD Cantonwine DE Chen YH Mukherjee B McElrath TF. Urinary phthalate metabolite and bisphenol A associations with ultrasound and delivery indices of fetal growth. Environ Int. (2016) 94:531–7. 10.1016/j.envint.2016.06.013 27320326 PMC4980186

[96] Casas M Valvi D Ballesteros-Gomez A Gascon M Fernandez MF Garcia-Esteban R. Exposure to bisphenol A and phthalates during pregnancy and ultrasound measures of fetal growth in the INMA-sabadell cohort. Environ Health Perspect. (2016) 124:521–8. 10.1289/ehp.1409190 26196298 PMC4829997

[97] Botton J Philippat C Calafat AM Carles S Charles M-A Slama R. Phthalate pregnancy exposure and male offspring growth from the intra-uterine period to five years of age. Environ Res. (2016) 151:601–9. 10.1016/j.envres.2016.08.033 27596487 PMC7950638

[98] Tsai YA Tsai MS Hou JW Lin CL Chen CY Chang CH. Evidence of high di(2-ethylhexyl) phthalate (DEHP) exposure due to tainted food intake in Taiwanese pregnant women and the health effects on birth outcomes. Sci Total Environ. (2018) 618:635–44. 10.1016/j.scitotenv.2017.07.175 29055577

[99] Philippat C Mortamais M Chevrier C Petit C Calafat AM Ye X. Exposure to phthalates and phenols during pregnancy and offspring size at birth. Environ Health Perspect. (2012) 120:464–70. 10.1289/ehp.1103634 21900077 PMC3295340

[100] Sathyanarayana S Barrett E Nguyen R Redmon B Haaland W Swan SH. First trimester phthalate exposure and infant birth weight in the infant development and environment study. Int J Environ Res Public Health. (2016) 13:945. 10.3390/ijerph13100945 27669283 PMC5086684

[101] Harley KG Berger K Rauch S Kogut K Claus Henn B Calafat AM. Association of prenatal urinary phthalate metabolite concentrations and childhood BMI and obesity. Pediatr Res. (2017) 82:405–15. 10.1038/pr.2017.112 28426647 PMC5581502

[102] Maresca MM Hoepner LA Hassoun A Oberfield SE Mooney SJ Calafat AM. Prenatal exposure to phthalates and childhood body size in an urban cohort. Environ Health Perspect. (2016) 124:514–20. 10.1289/ehp.1408750 26069025 PMC4829975

[103] Shoaff J Papandonatos GD Calafat AM Ye X Chen A Lanphear BP. Early-life phthalate exposure and adiposity at 8 years of age. Environ Health Perspect. (2017) 125:097008. 10.1289/EHP1022 28935615 PMC5915197

[104] Buckley JP Engel SM Mendez MA Richardson DB Daniels JL Calafat AM. Prenatal phthalate exposures and childhood fat mass in a New York city cohort. Environ Health Perspect. (2016) 124:507–13. 10.1289/ehp.1509788 26308089 PMC4829985

[105] Agay-Shay K Martinez D Valvi D Garcia-Esteban R Basagana X Robinson O. Exposure to endocrine-disrupting chemicals during pregnancy and weight at 7 years of age: a multi-pollutant approach. Environ Health Perspect. (2015) 123:1030–7. 10.1289/ehp.1409049 25956007 PMC4590760

[106] Buckley JP Engel SM Braun JM Whyatt RM Daniels JL Mendez MA. Prenatal phthalate exposures and body mass index among 4- to 7-year-old children: a pooled analysis. Epidemiology. (2016) 27:449–58. 10.1097/EDE.0000000000000436 26745610 PMC4821741

[107] Yang TC Peterson KE Meeker JD Sánchez BN Zhang Z Cantoral A. Exposure to bisphenol A and phthalates metabolites in the third trimester of pregnancy and BMI trajectories. Pediatr Obes. (2018) 13:550–7. 10.1111/ijpo.12279 29700996 PMC6988102

[108] Yu Z Han Y Shen R Huang K Xu Y-Y Wang Q-N. Gestational di-(2-ethylhexyl) phthalate exposure causes fetal intrauterine growth restriction through disturbing placental thyroid hormone receptor signaling. Toxicol Lett. (2018) 294:1–10. 10.1016/j.toxlet.2018.05.013 29753845

[109] Skakkebaek NE Rajpert-De Meyts E Buck Louis GM Toppari J Andersson AM Eisenberg ML. Male reproductive disorders and fertility trends: influences of environment and genetic susceptibility. Physiol Rev. (2016) 96:55–97. 10.1152/physrev.00017.2015 26582516 PMC4698396

[110] Wu S Zhu J Li Y Lin T Gan L Yuan X. Dynamic effect of di-2-(ethylhexyl) phthalate on testicular toxicity: epigenetic changes and their impact on gene expression. Int J Toxicol. (2010) 29:193–200. 10.1177/1091581809355488 20335514

[111] Arbuckle TE MacPherson S Barrett E Muckle G Séguin JR Foster WG. Do stressful life events during pregnancy modify associations between phthalates and anogenital distance in newborns? Environ Res. (2019) 177:108593. 10.1016/j.envres.2019.108593 31357157

[112] Huang PC Kuo PL Chou YY Lin SJ Lee CC. Association between prenatal exposure to phthalates and the health of newborns. Environ Int. (2009) 35:14–20. 10.1016/j.envint.2008.05.012 18640725

[113] Swan SH Main KM Liu F Stewart SL Kruse RL Calafat AM. Decrease in anogenital distance among male infants with prenatal phthalate exposure. Environ Health Perspect. (2005) 113:1056–61. 10.1289/ehp.8100 16079079 PMC1280349

[114] Swan SH Sathyanarayana S Barrett ES Janssen S Liu F Nguyen RH First trimester phthalate exposure and anogenital distance in newborns. Hum Reprod. (2015) 30:963–72. 10.1093/humrep/deu363 25697839 PMC4359397

[115] Suzuki Y Yoshinaga J Mizumoto Y Serizawa S Shiraishi H. Foetal exposure to phthalate esters and anogenital distance in male newborns. Int J Androl. (2012) 35:236–44. 10.1111/j.1365-2605.2011.01190.x 21696396

[116] Bustamante-Montes LP Hernandez-Valero MA Flores-Pimentel D Garcia-Fabila M Amaya-Chavez A Barr DB. Prenatal exposure to phthalates is associated with decreased anogenital distance and penile size in male newborns. J Dev Orig Health Dis. (2013) 4:300–6. 10.1017/S2040174413000172 24349678 PMC3862078

[117] Bornehag CG Carlstedt F Jonsson BA Lindh CH Jensen TK Bodin A. Prenatal phthalate exposures and anogenital distance in Swedish boys. Environ Health Perspect. (2015) 123:101–7. 10.1289/ehp.1408163 25353625 PMC4286276

[118] Wenzel AG Bloom MS Butts CD Wineland RJ Brock JW Cruze L. Influence of race on prenatal phthalate exposure and anogenital measurements among boys and girls. Environ Int. (2018) 110:61–70. 10.1016/j.envint.2017.10.007 29097052

[119] Swan SH. Environmental phthalate exposure in relation to reproductive outcomes and other health endpoints in humans. Environ Res. (2008) 108:177–84. 10.1016/j.envres.2008.08.007 18949837 PMC2775531

[120] Wagner-Mahler K Kurzenne JY Delattre I Berard E Mas JC Bornebush L. Prospective study on the prevalence and associated risk factors of cryptorchidism in 6246 newborn boys from nice area, France. Int J Androl. (2011) 34:e499–510. 10.1111/j.1365-2605.2011.01211.x 21831232

[121] Chevrier C Petit C Philippat C Mortamais M Slama R Rouget F. Maternal urinary phthalates and phenols and male genital anomalies. Epidemiology. (2012) 23:353–6. 10.1097/EDE.0b013e318246073e 22317818 PMC4724202

[122] Brucker-Davis F Wagner-Mahler K Delattre I Ducot B Ferrari P Bongain A. Cryptorchidism at birth in nice area (France) is associated with higher prenatal exposure to PCBs and DDE, as assessed by colostrum concentrations. Hum Reprod. (2008) 23:1708–18. 10.1093/humrep/den186 18503055

[123] Ormond G Nieuwenhuijsen MJ Nelson P Toledano MB Iszatt N Geneletti S. Endocrine disruptors in the workplace, hair spray, folate supplementation, and risk of hypospadias: case-control study. Environ Health Perspect. (2009) 117:303–7. 10.1289/ehp.11933 19270804 PMC2649236

[124] Vrijheid M Armstrong B Dolk H van Tongeren M Botting B. Risk of hypospadias in relation to maternal occupational exposure to potential endocrine disrupting chemicals. Occup Environ Med. (2003) 60:543–50. 10.1136/oem.60.8.543 12883014 PMC1740604

[125] Nassar N Abeywardana P Barker A Bower C. Parental occupational exposure to potential endocrine disrupting chemicals and risk of hypospadias in infants. Occup Environ Med. (2010) 67:585–9. 10.1136/oem.2009.048272 19939854

[126] Jensen TK Frederiksen H Kyhl HB Lassen TH Swan SH Bornehag CG. Prenatal exposure to phthalates and anogenital distance in male infants from a low-exposed danish cohort (2010-2012). Environ Health Perspect. (2016) 124:1107–13. 10.1289/ehp.1509870 26672060 PMC4937858

[127] Arbuckle TE Agarwal A MacPherson SH Fraser WD Sathyanarayana S Ramsay T. Prenatal exposure to phthalates and phenols and infant endocrine-sensitive outcomes: The MIREC study. Environ Int. (2018) 120:572–83. 10.1016/j.envint.2018.08.034 30195175

